# Traumatic Floating Clavicle: A Case Report and Literature Review

**DOI:** 10.1155/2013/386089

**Published:** 2013-12-04

**Authors:** Mohamad Gouse, Korula Mani Jacob, Pradeep Mathew Poonnoose

**Affiliations:** Department of Orthopaedics, Christian Medical College, Vellore 632004, India

## Abstract

Bipolar fracture dislocations of the clavicle are rare injuries, usually the result of high-energy direct trauma. Since the original description by Porral in 1831, only a handful of individual case reports and case series by Beckman and Sanders have been reported in the literature. Management of these injuries has remained controversial ranging from nonoperative to aggressive surgery. We report on the case of a young army cadet who had a fracture of the lateral end of the clavicle, with an anterior dislocation of the sternoclavicular joint. Despite being planned for surgery, at the patients request, it was decided to manage the lesion conservatively with graded physiotherapy. At one-year follow-up, he had full pain-free, functional range of movement of the shoulder. This young high demand patient had a good outcome with conservative management, despite going against the current trend towards surgical treatment. We present this case with a review of the literature, highlighting the various management options for this rare lesion.

## 1. Introduction

While fractures of the clavicle and isolated dislocations of the acromioclavicular (AC) joints are commonly encountered in clinical practice, bipolar fracture dislocations of the clavicle are rare. This rare injury refers to simultaneous injuries to the medial and lateral end of the clavicle and has been called variously—complete dislocation [1], bipolar dislocation [2], panclavicular dislocation [3], bifocal clavicular dislocation [4], and traumatic floating clavicle [5]. Bipolar fracture dislocations of the clavicle are rare injuries, usually the result of high-energy trauma [4, 6, 7]. Since the original description by Porral in 1831 [8], only a handful of individual case reports and the case series of Beckman [1] and Sanders et al. [9] have been reported in the literature. Management of these injuries has remained controversial ranging from nonoperative [10] to aggressive surgery [11]—even to the extent of total clavicle excision for a neglected dislocation [2]. It is noted through a careful review of the literature that surgical options have been preferred for younger and more active patients whereas the older and more sedentary patients were managed nonoperatively [4, 5]. We report a case of traumatic “floating clavicle” in a young, high demand patient, which was managed conservatively, in contrast to surgical treatment that has been previously recommended in the literature.

## 2. Case Report 

**Figure 1:**
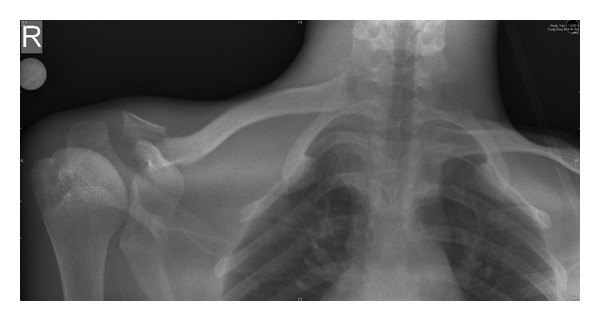
Radiograph at first presentation showing fracture lateral end of right clavicle and sternoclavicular dislocation—the floating clavicle.

**Figure 2:**
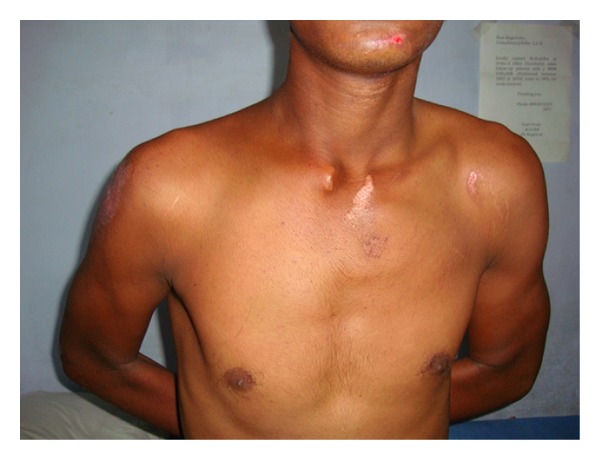
Clinical photograph showing cosmetic deformity but full internal rotation at 18-month follow-up.

**Figure 3:**
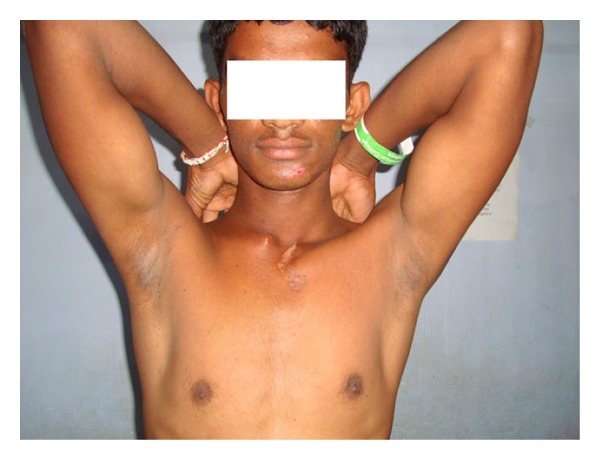
Clinical photograph showing full range of shoulder abduction at 18-month follow-up.

**Figure 4:**
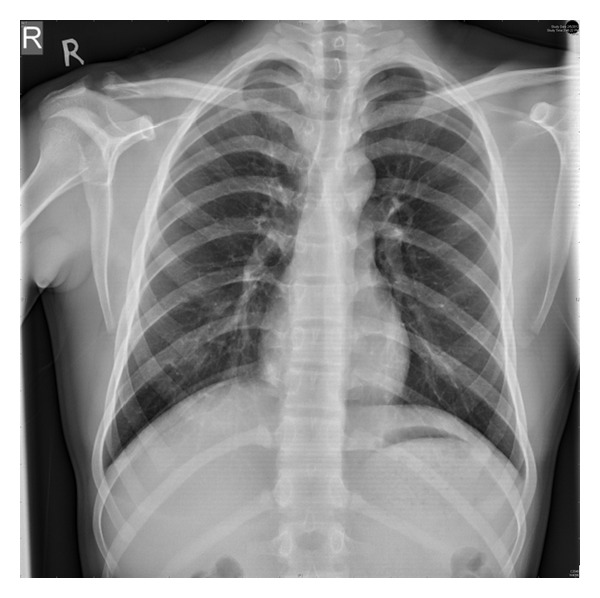
Follow-up radiograph at 18 months showing union at the lateral end of the clavicle and persistent sternoclavicular dislocation.

A 19-year-old army cadet presented to our emergency department with a painful shoulder following a fall off a motor bike. He had multiple abrasions over the face and right shoulder region and had difficulty lifting his upper limb. There was tenderness and crepitus at the lateral end of the clavicle, with an obvious anterior dislocation of the sternoclavicular joint as noted by a swelling over the medial end of the clavicle and sternoclavicular joint. There were no distal neurovascular deficits. Plain radiographs revealed a displaced fracture of the lateral end of clavicle and an anterior dislocation of the sternoclavicular joint (Figure 1). The patient was not keen on surgical intervention, and hence it was decided to treat the patient conservatively. He was advised to use a clavicle brace and broad arm sling for a period of 4 weeks followed by assisted pendulum exercises and gravity eliminated range of movement exercises. Despite the advice to proceed cautiously with physiotherapy, he removed the sling after 2 weeks and started performing all his normal daily activities with his right upper limb. He failed to make his outpatient appointment at 4 weeks and rejoined service in the army within a month. When he presented to us 18 months after his injury, though he had a mild deformity over the clavicle, (Figure 2) he had no functional disability (Figures 2 and 3). Plain radiographs at final follow-up showed a persistent sternoclavicular dislocation with union of the lateral end of clavicle fracture (Figure 4). DASH score at 18-month follow-up was zero (no disability). The range of motion in the shoulder was normal with full range of abduction, elevation, and rotations. He was able to perform 100 pushups, throw objects over long distances, and use a combat rifle without pain. He had no difficulty performing any of the activities that were required of him in the army.

## 3. Discussion

Bipolar fracture dislocations of the clavicle refer to simultaneous injuries to the medial and lateral end of the clavicle and its related articulations at the coracoclavicular and acromioclavicular joints. Synonyms for this injury include complete dislocation [1], bipolar dislocation [2], panclavicular dislocation [3], bifocal clavicular dislocation [4], and traumatic floating clavicle [5]. These injuries are rare, and there has been no definitive incidence of these injuries reported in the literature. However, to put it in perspective, in a series of 614 clavicle fractures, only 0.8% had segmental injuries—the closest approximation to bipolar injury [12]. Since the original description by Porral in 1831 [8], only a handful of individual case reports and the case series of Beckman [1] and Sanders et al. [9] have been reported in the literature.

The mechanism of injury is thought to be the result of high-energy trauma. It has been hypothesized that, for these segmental injuries to occur, the clavicle has to be subjected to two separate, but sequential forces [4, 6, 7]—a medially directed blow to the lateral aspect of the shoulder, and an injury associated with torsion of the trunk [3, 13]. It is postulated that the clavicle acts as a lever, anchored at the conoid and trapezoid corcoclavicular (CC) ligaments. There is an initial anterior subluxation of the sternoclavicular joint, which may be anterosuperior [3, 4], or in some cases antero-inferior [14, 15]. This is followed by posterior subluxation of the AC joint or fracture of the lateral end of clavicle, with the CC ligament acting as a fulcrum for the clavicle. The conoid and trapezoid CC ligaments may be preserved [14].

As bipolar injury of the clavicle is a rarity, there is not much evidence-based literature on the best method of treatment. Treatment modalities include nonsurgical options such as attempts at reduction and the use of a plaster cast, figure of eight harness [3], and arm slings [4, 5]. Surgical treatment includes the use of Kirschner wires and screws [14, 16], hooked plates [15], reconstruction plates [17], tension bands [18], and ligamentous reconstruction [13, 14, 18].

A review of the literature would suggest that there are 2 groups of patients and that the treatment should be based on the age and the activity demand of the patient. The older, more sedentary patient tended to be treated nonoperatively, whereas young active patients were more commonly subject to surgical intervention. A comprehensive review of the published literature on the various treatment options used by different authors is shown in Table 1.

Eni-Olotu and Hobbs treated a 63-year-old lady with floating clavicle conservatively with a sling, and the patient had a relatively good functional outcome [4]. Jain treated a 77-year-old man who had sustained a similar injury with a simple arm sling. At 6 weeks, he had good range of movement of the shoulder. He concluded that surgical treatment was not necessary [5]. There were many more examples of similar treatment in the literature as seen in Table 1 for the low demand patient. Literature suggests conservative treatment for the elderly and patients with low functional demands.

Schuh et al. surgically treated a 23-old-year boy with bipolar dislocation with tension band wiring at both ends of the clavicle. He recommended surgical management for young patients with high physical demands [18]. Arenas et al. treated a 26-year-old male with kirschner wire fixation of both the AC and SC joints, and the patient had a good functional and radiological outcome [14]. Scapinelli used a transarticular Kirschner wire for the sternoclavicular joint, and a tension band wiring for the acromioclavicular joint [13]. Surgical reduction and fixation of both AC and SC joint seem to be the choice of most surgeons who have encountered the lesion in the young, high demand patient.

Our young army cadet did not have any functional disability despite nonoperative management of his injury. We suggest that conservative management with a sling for a few weeks should suffice even for young patients with high physical demands. We suggest that surgical intervention can be reserved for any complications that might arise subsequently.

## 4. Conclusion

Though the limited body of the literature available on bipolar clavicular dislocations suggests surgical management in the young active individual and conservative management for the older individual, the observed good results in our cadet seem to suggest that full functional recovery is possible in a high demand young individual, even with nonoperative management.

## Figures and Tables

**Table 1:** Review of the existing literature on floating clavicle.

No.	Author	Year published	No. of patients	Age of patients	Treatment modality
1	Gearen and Petty [3]	1982	1	27	Surgical
2	Jain [5]	1984	1	77	Conservative
3	Cook and Horowitz [19]	1987	1	60	Conservative
4	Echo et al. [16]	1988	1	20	Surgical
5	Thomas and Friedman [17]	1989	1	28	Surgical
6	Sanders et al. [9]	1990	6	26, 35, 20, 41, 67, 21	4 surgical 2 conservative
7	Gaudernak and Poigenfurst [20]	1991	1	17	Surgical
8	Arenas et al. [14]	1993	1	26	Surgical
9	Tanlin [21]	1996	1	19	Surgical
10	Eni-Olotu and Hobbs [4]	1997	1	63	Conservative
11	Le Huec et al. [11]	1998	2	58	Surgical
				18	
12	Akpinar et al. [22]	2002	1	55	Surgical
13	Scapinelli [13]	2004	1	18	Surgical
14	Argintar et al. [23]	2011	1	20	Surgical
15	Pa$\overset{ˇ}{\text{s}}$a and Kalandra [24]	2011	1	17	Surgical
16	Yurdakul et al. [25]	2012	1	71	Conservative
17	Argintar et al. [23]	2011	1	55	Surgical
18	Sethi et al. [26]	2012	1	32	Surgical
19	Schuh et al. [18]	2012	1	23	Surgical
20	Jiang et al. [27]	2012	1	21	Surgical
21	Dudda et al. [28]	2012	1	70	Conservative
22	Daolagupu et al. [29]	2013	1	41	Surgical
23	Dudda et al. [28]	2013	1	60	Conservative
24	Daolagupu et al. [29]	2013	1	12	Surgical
